# Coffee and Tea

**Published:** 1858-07

**Authors:** 


### COFFEE AND TEA.

OUR readers know that we consider the regular and moderate use of tea and coffee, for breakfast and supper, a healthful luxury. The arguments we have offered before, the safe method of using these articles, may be repeated; they should be taken hot, with or without cream or sugar, only at meal-times, and never increasing them, in frequency, quantity, or strength. By attention to these rules, they may be taken daily for a lifetime, with daily advantage and comfort. There are persons who are rendered uncomfortable in some way by the use of one or both of them; but no doubt this has been brought about by their extravagant employment. Where they evidently disagree with a person, they should be discontinued, at least for a time. When tea or coffee in moderate quantities cause any uncomfortable sensations, it is proof of a disordered digestion; and when that is corrected, these beverages may be drank with their old-time impunity.

But if coffee is good at all, it is best in all its purity—as gold is best without alloy—as honey is best with all its sweetness—as perfumes are best before they have lost their strength. All know that the best method of making soup is to retain the essence of the meat, by boiling it in a closed vessel. And it is certainly a phenomena of mind, that this self-evident principle in physics, a principle so generally known to persons of even moderate intelligence, should have been lost sight of by every body, and for a time as long as since the introduction of coffee as a common beverage. Every day in the last half century, in millions of families, coffee has been prepared by boiling away its most volatile qualities, the refuse only being drunk—to say nothing of the useless waste.

ARTHUR, BURNHAM & GILROY, of Philadelphia, manufacture *The Old Dominion Coffee Pot;* and it is amply sufficient to state, that the *aroma* is preserved; and that the longer it is boiled, the better is the coffee, even if kept boiling for two hours. The pots are of all sizes, from one quart to sixteen, and can be made still larger; and it is claimed that they give good coffee, at one fourth less cost, than by the old method of boiling. We commend the *Old Dominion Coffee Pot* to all lovers of good coffee, as we personally know that it is one of the few "*new things*" offered to the public, in which no imposition is practiced, and which has the double vouchers of science and common sense, at the cost of two dollars.

The same house has other articles of domestic economy and comfort; but as their prices are not named, we do not mention them. We never could understand why persons who have houses, lots, lands, goods, books, or property of any description, to sell, should not state plainly the price. It is so evidently the dictate of common sense, that the price of anything for sale should be known, that we take it for granted, when a man offers anything for sale, and does not tell the price, the information is suppressed for the advantage of the seller. Our JOURNAL is for practical purposes, to benefit its subscribers, and we intend to notice no book, and admit no advertisement of any necessary domestic article, whose price is not influenced by the seasons, unless the price is distinctly named. More than two years ago, we suggested this to publishers; but it is scarcely ever attended to, except by the straight-forward practical house of FOWLER & WELLS. Publishers need send us no book to notice, unless the price is stated for which it will be sent post-paid to our subscribers. A man lives in Louisiana; a book is noticed; he wants to know its price; writes for the information to us: we go a journey of two miles and back to the publishers; write a reply; the money is sent; we repeat the journey; and the man gets his book at an extra charge of three postages and stationery, four omnibus rides, amounting to about forty cents, which is, sometimes, more than the cost of the book, to say nothing of the delay to the purchaser, of the advantages of the nimble penny to the seller, and, more than all, of the loss of two hours and a half to the friend whose courtesy is so largely taxed to make the inquiry—and taxed with as much indifference as if it involved "going down town" of a village nine rods long, and no rods broad.

Will our country cousins and some others remember, that the smallest request they can make as to the price or practicability of anything sold in New York, cannot be ascertained by us, except on chance occasions, short of a five-mile ride or walk, and an hour's time.

